# Exploring dynamic protein-protein interactions in cassava through the integrative interactome network

**DOI:** 10.1038/s41598-020-63536-0

**Published:** 2020-04-16

**Authors:** Ratana Thanasomboon, Saowalak Kalapanulak, Supatcharee Netrphan, Treenut Saithong

**Affiliations:** 1 Biological Engineering Program, Faculty of Engineering, King Mongkut’s University of Technology Thonburi, Bangkok, 10140 Thailand; 2 Center for Agricultural Systems Biology, Systems Biology and Bioinformatics Research Group, Pilot Plant Development and Training Institute, King Mongkut’s University of Technology Thonburi (Bang Khun Thian), Bangkok, 10150 Thailand; 3 Bioinformatics and Systems Biology Program, School of Bioresources and Technology, King Mongkut’s University of Technology Thonburi (Bang Khun Thian), Bangkok, 10150 Thailand; 4 National Center for Genetic Engineering and Biotechnology, Pathum Thani, 12120 Thailand

**Keywords:** Computational biology and bioinformatics, Data integration, Systems biology, Dynamic networks

## Abstract

Protein-protein interactions (PPIs) play an essential role in cellular regulatory processes. Despite, in-depth studies to uncover the mystery of PPI-mediated regulations are still lacking. Here, an integrative interactome network (MePPI-Ux) was obtained by incorporating expression data into the improved genome-scale interactome network of cassava (MePPI-U). The MePPI-U, constructed by both interolog- and domain-based approaches, contained 3,638,916 interactions and 24,590 proteins (59% of proteins in the cassava AM560 genome version 6). After incorporating expression data as information of state, the MePPI-U rewired to represent condition-dependent PPIs (MePPI-Ux), enabling us to envisage dynamic PPIs (DPINs) that occur at specific conditions. The MePPI-Ux was exploited to demonstrate timely PPIs of cassava under various conditions, namely drought stress, brown streak virus (CBSV) infection, and starch biosynthesis in leaf/root tissues. MePPI-Ux_drought_ and MePPI-Ux_CBSV_ suggested involved PPIs in response to stress. MePPI-Ux_SB,leaf_ and MePPI-Ux_SB,root_ suggested the involvement of interactions among transcription factor proteins in modulating how leaf or root starch is synthesized. These findings deepened our knowledge of the regulatory roles of PPIs in cassava and would undeniably assist targeted breeding efforts to improve starch quality and quantity.

## Introduction

In cells, protein-protein interaction (PPI) is an important step that mediates the action of expressed proteins to function precisely in the regulatory process of signal transduction, homeostasis, and organ formation^1^. Over 60 percent of entire proteins in genomes need to interact with their counterparts to achieve their functions, usually through post-translational modification (PTM)^2^. The interaction between proteins might last lasting as in cases of stable multi-protein complexes. These interacting proteins are often found in cellular structures, e.g. binding of actin-cross-linking protein (CROLIN1) and F-actin protein to form actin structures in Arabiodopsis^3^, and are involved in the process of cell and organ formation, e.g. heterodimeric complex of the catalytic molybdopterin subunit and a c-type cytochrome subunit in *Starkeya novella* involved in electron transfer of sulfite-oxidizing enzyme^4^. The interactions could also be temporary, allowing transient mediation of regulatory states through changes in protein activity, stability, and localization across cellular compartments^5^, which are sources of dynamic regulation in cells. Some examples of transient protein interactions are the phosphorylation-dependent function of starch branching enzyme IIa (SBEIIa) in wheat, whereby the active form of SBEIIa is modulated by its interaction with kinase or phosphatase proteins;^6^ the stability of autophagy protein 6 (ATG6) in Arabidopsis, which depends on its interaction with tumor necrosis factor receptor-associated factor TRAF1a and TRAF1b proteins;^7^ and CSN1-induced COP1 nuclear localization in Arabidopsis, where the association of COP1 and signalsome COP9 (CSN) is crucial^8^. Monitoring the transient interaction of proteins has been a real challenge; thus, measurement technologies such as combinatorial blue native PAGE and mass spectrometry^9^, mass spectrometry^10^, NMR spectroscopy^11^, bimolecular fluorescence complementation^12^, label-free biosensor^13^, and yeast two-hybrid (Y2H)^14^ have been developed to capture such interactions, but these are time consuming and expensive.

Computational prediction techniques have recently been employed to facilitate the identification of PPIs. Inference-based techniques rely on the knowledge from well-studied organisms, existing in literature, and thermodynamic properties of protein interactions. Interolog^15^ and domain-based^16^ methods, which predict unknown PPIs based on the evidence of interactions between their orthologous proteins and the presence of interacting protein domains, respectively, are the most widely techniques^16–25^. The first interactome network of plant species studied in Arabidopsis inferred 19,979 putative PPIs from 73,454 PPIs, determined experimentally in yeast, nematode worm, fruit fly, and humans^20^. Later, interactome network of rice was extendedly inferred from both experimental and prediction data^23^. Additionally, advanced algorithms in modern deep learning approaches have been introduced to analyze large-scale data, to provide *ab-initio* predictions^26–32^. With these approaches, PPI networks in various organisms^15,20,33,34^ have been constructed, but these are neither time nor condition-specific; thus, they do not reflect the dynamics of PPIs participating in the cellular response to exposed environments. Attempts to investigate the transient PPIs that introduce changes in the regulatory process in response to prevailing conditions have been made through the integration of state-dependent data into the interactome networks. For example, Lichtenberg *et al*. (2005)^35^ and Wang *et al*. (2013)^36^ constructed dynamic PPI networks of the mitotic cell cycle in yeast using time series microarray data, based on the assumption that PPIs occur or function only if the proteins are present or expressed under the studied conditions. This integrative method proposed a series of PPI subnetworks functioning in each condition and provided a state-specific PPI network governing the yeast cell cycle.

In plants, PPIs are important for the development^37,38^ and stress response processes^39,40^. Plenty of evidence shows that PPIs modulate metabolic regulation affecting the yield of crop plants. For example, the rice 14-3-3 protein complex can interact with FD transcription factors to form a florigen activation complex (FAC) which affects yield through promoting the flowering pathway^41^. Another report in tomato shows a disruption of the FAC complex by an imbalance between flower-promoting (SFT) and flower-repressing (SP) signals, due to perturbation, is related to yield^42^. Several PPI networks have been reported for various species, including Arabidopsis^21,22,43–45^, rice^17,23,46^, maize^24^, tomato^25^, and sweet orange^47^. These were mainly based on the interolog or domain-based approach, though a combination of both was used to gain more prediction coverage in genome-scale studies. With this success, the next challenge is to access the time- and/or condition-specific interactions of proteins that might be the causes of various plant phenotypes.

Cassava (*Manihot esculenta* Crantz) is a staple crop whose starchy roots feed at least 800 million people annually^48^. Cassava yield and production are, thus, crucial for securing food sufficiency worldwide. To elucidate cellular regulations involved in starch biosynthesis as well as crop yield, protein expression in various conditions, for example during root development^49^, drought stress^50^, and in specific tissues^51–54^, has been studied. However, the interaction among these expressed proteins, which is believed to be key to the regulatory processes underlying observed phenotypes, is still unclear. Qin *et al*. (2017)^55^ proposed a group of cassava PPIs (196 interactions of 76 proteins) that are potentially associated with post-harvest physiological deterioration (PPD) of roots, based on the interactome network of Arabidopsis. Later, the first genome-wide interactome network of cassava, named MePPI-In, was proposed by Thanasomboon *et al*. (2017)^56^, and this provides the platform for this current study. MePPI-In was developed based on the interolog method and consists of 90,173 PPIs and 7,209 proteins, but it is not able to elaborate on PPIs functioning in a particular condition. Herein, we propose an integrative interactome network (MePPI-Ux) that could be used to capture the dynamic PPIs that are linked to different regulatory processes in cassava. The genome-scale PPI network of cassava (MePPI-In) was improved by expanding the number of template species for the interolog-based prediction and by increasing the coverage of prediction with the domain-based approach, using the updated genome sequence (v.6). The resulting network, so called MePPI-U, consisted of 3,638,916 interactions and 24,590 proteins covering 59 percent of proteins in the cassava genome. The MePPI-U provides a greater percent coverage than its precursor, MePPI-In^56^ (hereafter called MePPI-In_4_^56^). Gene and protein expression data were incorporated into MePPI-U as information of state, enabling it to represent the PPIs under the specific conditions (MePPI-Ux). The MePPI-Ux was exemplified to investigate condition-dependent PPIs under drought stress, cassava brown streak virus (CBSV) infection, and starch biosynthesis in leaf and root tissues. The MePPI-Ux,_CBSV_ and MePPI-Ux,_drought_ provided insights into PPI modulated stress response, whereas MePPI-Ux_SB,leaf_ and MePPI-Ux_SB,root_ suggested that starch biosynthesis in these individual tissues might be mediated by the interaction of transcription factor proteins. These findings supported the rationale of the integrative interactome network MePPI-Ux in deepening the study of PPIs under the prevailing conditions.

## Methods

### Protein-protein interaction network construction

**Figure 1:**
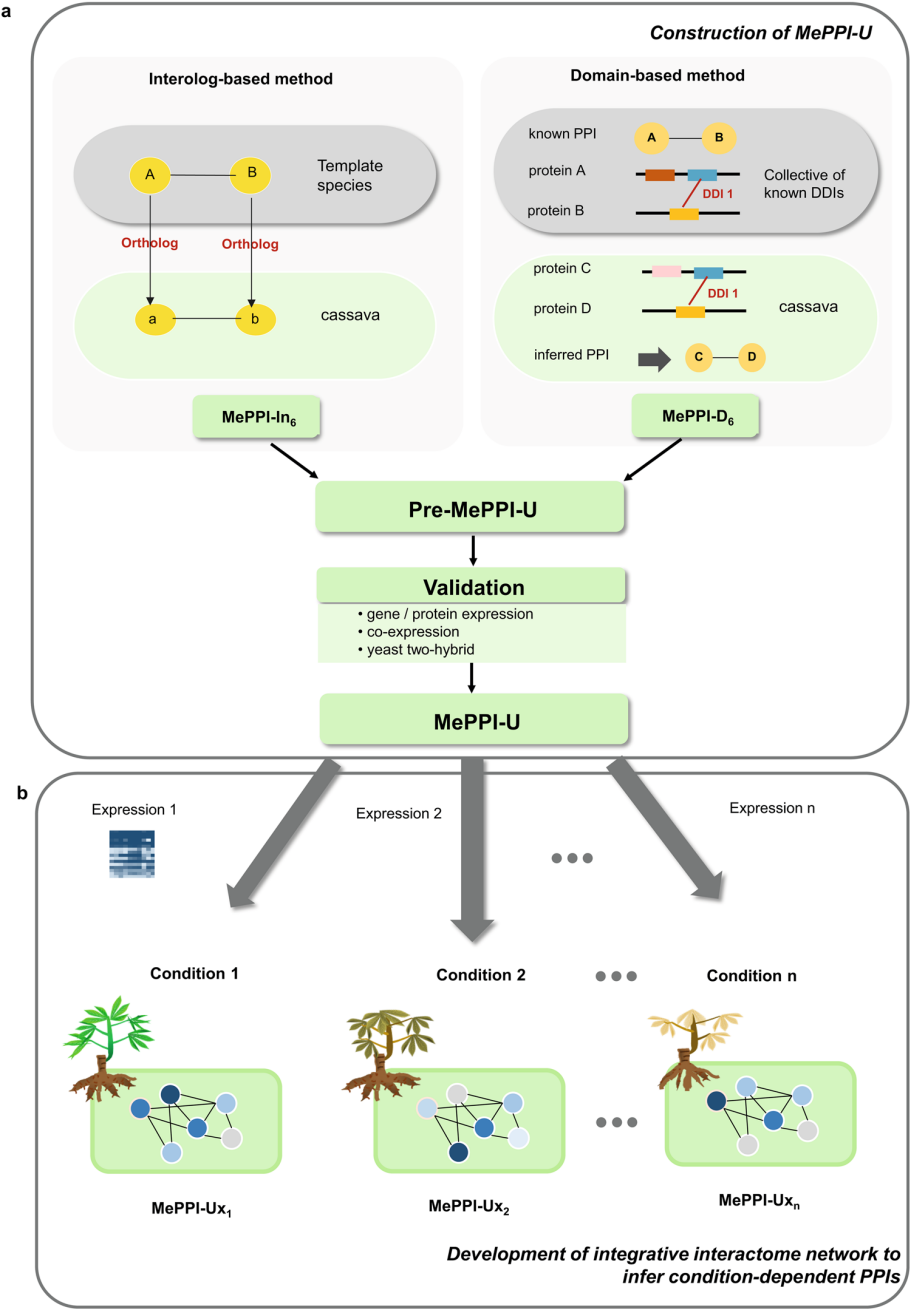
Overall methodology of PPI prediction in cassava consisting of two parts: (**a**) construction of protein-protein interaction network of cassava (MePPI-U) using interolog-based and domain-based approaches and (**b**) development of integrative interactome network to infer PPIs acting under various conditions.

The interolog-based and domain-based approaches were employed to construct the cassava protein-protein interaction network, called hereafter MePPI-U. First, the interactions of proteins were predicted based on the interolog method whereby PPIs of cassava were inferred from their orthologs in nine template organisms, selected based upon these criteria: (1) having a close evolution with cassava (i.e. *Ricinus communis* (castor bean) and *Populus trichocarpa* (poplar)), (2) being a starch-storing plant (i.e. *Solanum tuberosum* (potato), *Zea mays* (maize), and *Oryza sativa* (rice)), or (3) having abundant PPI information (i.e. *Arabidopsis thaliana*, *Lycopersicum solanaceae* (tomato), *Glycine max* (soybean), and *Citrus sinensis* (sweet orange)). Their protein information was retrieved from Phytozome V.11^57^, and the interactions were exhaustively collected from ten PPI databases, AtPID^21^, AtPIN^22^, PRIN^23^, PPIM^24^, PTIR^25^, APID^43^,PAIR^45^, IntAct^58^, MINT^59^, and Ding *et al*.^47^. The orthologous proteins of cassava were identified by BLASTp sequence alignment based on these criteria: percentage of identity ≥ 60%, percentage of coverage ≥ 80% and e-value ≤10^−10^. PPIs inferred by the interolog-based method were then used to construct a network, denoted as MePPI-In_6_. In parallel with the MePPI-In_6_, MePPI-D_6_ was constructed based on domain information. The PPIs were predicted when binding domains of both proteins interact. The information on cassava protein domain and domain-domain interaction (DDI) was gathered from Pfam^60^ and iPfam^61^ databases, respectively. In this work, the protein-protein interaction was predicted when, at least, one DDI occurs between protein pairs. PPIs inferred by the DDI-based method were used to construct a network denoted as MePPI-D_6_. Third, the resulting PPIs from both interolog- and domain-based predictions (MePPI-In_6_ and MePPI-D_6,_ respectively) were combined to generate the MePPI-U, a network with all possible PPIs of cassava. The overall framework for MePPI-U construction is described in Fig. 1.

### Supporting MePPI-U with expression data

The constructed MePPI-U was validated on the fact that interaction of proteins could be achieved only if proteins or the corresponding protein-coding genes are expressed and with high probability if the expression patterns are correlated. Here, 15 expression datasets were employed to support the existence of proteins in MePPI-U. From these datasets, seven represent protein expression in cassava roots (fibrous and storage roots), leaves, embryos and plantlets at different development stages^49–54,62^ and eight are gene expression in the form of either microarray-based datasets^63–66^ or RNA-seq-based datasets^67–70^. Subsequently, co-expression patterns of the interacting protein pairs were determined using information from five time-series expression datasets of Naconsie *et al*.^49^, An *et al*.^63^, Li *et al*.^64^, Amuge *et al*.^69^ and Wang *et al*.^70^. The co-expression analysis was conducted only for highly expressed genes or proteins that exhibit expression levels greater than 80 percentile rank to ensure the existence of the interacting proteins. Correlation of expression profiles was determined by Pearson’s statistics; the profiles were adjudged correlated when the Pearson correlation coefficient ($PCC$) is> 0.90 for microarray-measured data, or> 0.99 for RNA-seq measured data, at *p*-value < 0.1.

### Validation of MePPI-U with PPIs from yeast two-hybrid method

Putative PPIs in MePPI-U were validated based on the results from yeast two-hybrid method. Here, 200 investigated interactions (47 interactions and 153 non-interactions) between eight CBLs (Calcineurin B-like (CBL) protein) and 25 CIPKs (CBL-interacting protein kinase) proteins from MePPI-U were compared with the results from yeast two-hybrid reported by Mo *et al*. (2018)^71^. The predictive power for PPIs in MePPI-U was measured using a confusion matrix^72^, whereby accuracy, precision, specificity and sensitivity were determined.

### Determination of confidence score

The confidence of the predicted PPIs in MePPI-U was evaluated based on the agreement of predictions from both methods: interolog and domain-domain interaction analyses. Unlike our previously constructed network “MePPI-In_4_”^56^, a domain-based prediction was performed to extend the search for putative PPIs in cassava. The confidence value ($CV$) score was calculated to represent the amount of information supporting the prediction of each PPI. To suit the objective of measurement, the original formula for the calculation of $CV$ score^56^ was modified accordingly. Here, the confidence score was a mean of the interolog ($C{V}_{interolog}$)^56^ and DDI ($C{V}_{DDI}$)^56^ confidence values (Eq. 1). The confidence score of the interolog-based prediction, $C{V}_{interolog}$, was calculated based on the number of plant templates from which the cassava PPI was inferred and also the source of identified interactions (i.e. computational prediction or experimental measurement) in template species (Eq. 2). The confidence score of the domain-based prediction, $C{V}_{DDI}$, was determined based on the number of domain-domain interactions (DDIs) underlying the prediction of a protein pair (Eq. 3).

1 $$CV=\frac{C{V}_{interolog}+C{V}_{DDI}}{2}$$

2 $$C{V}_{interolog}=\frac{{\sum }_{i=1}^{N}{S}_{i}{M}_{i}}{N}$$

3 $$C{V}_{DDI}=dD$$

$S\in \{0,1\}$ is the existence factor representing the occurrence of the orthologous protein pairs in the cassava genome. $M$ refers to the reliability of the protein-protein interaction regarding the inference methods as evidenced in plant templates, $0.5$ for computational prediction, and $1$ for experimental measurement. $N\in \{1,\,2,\ldots ,9\}$ is the number of species from which the protein-protein interactions in cassava were inferred. $D=[0,\,1]$ refers to domain enrichment, which is defined as the ratio of predicted DDIs to all possible interactions that could happen among identified domains in a protein pair. $d\in \{0.5,1\}$ is the correction factor of *D* to compensate the bias caused by varying number of domains in each protein pair, 0.5 for interactions of a single domain protein, and 1 for otherwise.

### Network topology and functional analysis

The topology of the interactome network was analyzed using network analyzer plugin tools in Cytoscape^73^. The scale-free property of the predicted network was examined and compared with other published plant PPI networks^22,23^. The biological functions of proteins in MePPI-U were investigated based on the ontology of related genes. GO enrichment analysis was performed through AgriGO^74^ and visualized by REVIGO^75^. The enriched functions were proposed based on hypergeometric statistics with *p*-value <0.05 and presented in terms of biological processes, molecular functions and cellular components.

### Transcriptome data analysis and integration

To investigate the condition-dependent PPIs, the integrative interactome network, MePPI-Ux, was constructed by incorporating transcriptome data into MePPI-U as to infer expression of the encoding proteins. RNA-seq datasets of gene expression under cold and drought stress^63^, viral infection^69^ and in leaf/root tissues^67^, in cassava, were employed to study the PPI network. The gene expression in each dataset was ranked by percentile to standardize the data. Only genes with an expression level greater than the bottom 10^th^ percentile of entire genes in the dataset were integrated into MePPI-U to infer the putative PPIs occurring in that particular condition. The expression of proteins in MePPI-Ux was classified based on the percentile into five levels: ≥ 90 percentiles, 75 ≤ percentile <90, 50 ≤ percentile <75, 25 ≤ percentile <50 and 10 <percentile <25, from high percentile (dark blue) to low percentile (light blue).

## Results and Discussion

### Cassava protein-protein interaction network (MePPI-U)

The genome-wide PPI network of cassava was exhaustively constructed from the known PPIs from other plant species and available domain-domain interaction data using interolog-based and domain-based methods. For the interolog-based method, cassava PPIs were inferred from nine well-studied plants, namely Arabidopsis, rice, maize, potato, tomato, sweet orange, poplar, castor bean and soybean. The plant species that contained the most abundant was maize (2,762,560 PPIs), followed by tomato (357,946 PPIs), Arabidopsis (235,215 PPIs), rice (76,829 PPIs), sweet orange (13,852 PPIs), potato (52 PPIs), castor bean (10 PPIs), soybean (10 PPIs) and poplar (8 PPIs) (Table S1). These differed in comparison with the previous work^56^, in which Arabidopsis provided the most PPI information. These data were employed to predict PPIs in cassava based on the functional conservation assumption of the orthologous proteins. Cassava orthologous proteins of each known PPI were searched by Blastp based on the following criteria: identity ≥ 60%, coverage ≥ 80%, and E-value ≤ 10^−10^. The results showed that most inferred PPIs were from Arabidopsis (107,235 PPIs), followed by tomato (97,885 PPIs), maize (32,894 PPIs), rice (17,697 PPIs), sweet orange (2,647 PPIs), potato (17 PPIs), soybean (7 PPIs), poplar (5 PPIs) and castor bean (1 PPI). The majority of putative PPIs (~ 95%; 236,008 PPIs) were predicted from the interactions present in, at most, one of nine templates used (Fig. S5); 10,234 PPIs (~ 5%) were from 2-5 organisms, 8,883 PPIs by two, 1,178 PPIs by three, 161 PPIs by four, and 12 PPIs by five organisms. All inferred cassava PPIs were subsequently combined to represent the cassava PPI network based on the interolog method (MePPI-In_6_). The resulting MePPI-In_6_ network, as described in Table S1, consisted of 246,242 PPIs and 13,766 proteins (33 percent of proteins in the cassava genome). This coverage of proteins in MePPI-In_6_ was 12 percent greater than that of MePPI-In_4_^56^, its counterpart from a previous work that used the same method. Two main reasons for this difference are updates of the cassava genome database and PPI information of plant templates. The genome update resulted from re-sequencing and re-annotation of genes and proteins, leading to different information. Comparing proteins from both genome versions shows that around 52% of the proteins are similar, while 48% have different information (Fig. S2b). In addition, increases in the number of plant templates (7 in MePPI-In_4_^56^ and 9 in this work) and the number of PPIs, particularly from maize (25 in the MePPI-In_4_^56^ and 2,762,560 in this work) offer more chance to predict cassava PPIs (MePPI-In_6_). However, prediction by this method seems dependent on the phylogenetic relatedness of cassava to the template species, as shown by the higher number of MePPIs predicted from Arabidopsis (107,235 PPIs), a dicot like cassava, than from maize (32,894 PPIs), a monocot. Figure S2 shows that ~9.2% (22,730) of PPIs in MePPI-In_6_ are present in MePPI-In_4_;^56^ MePPI-In_6_ has more unique PPIs (223,512) than MePPI-In_4_^56^ (67,443), most likely due to the use of different cassava genomes.

Since physical binding of proteins usually occurs via domain affinity, the information on domain-domain interaction (DDI) was exploited to predict interactions between cassava proteins beyond the current knowledge of PPIs in template species. Basically, two proteins are expected to be able to interact if they contain interacting domains. To infer interactions of proteins based on DDI information, the entire proteins encoded in the cassava genome were searched for the presence of domains, using information from the Pfam database (https://pfam.xfam.org). The analysis indicated that 30,025 of 41,381 proteins (~ 73 percent of proteins in the genome) from the database had at least one functional domain, which enabled them to physically interact with their counterparts. The interactions of those proteins were subsequently predicted based on information on interacting domains from the iPfam database (http://ipfam.org). The database version used in this study, as updated in 2016, classified DDIs into three classes: inter-chain (the DDI between different polypeptide chains), intra-chain (the DDI within a single polypeptide chain), and both; in comparison, the older version employed for MePPI-In_4_^56^ only contained DDI information without classification. In this study, only the inter-chain DDIs were used to predict the interactions between proteins. The domain-based PPI prediction of cassava, denoted as MePPI-D_6_, consisted of 3,424,602 PPIs interconnecting 20,142 proteins or 49 percent of proteins in the cassava genome.

**Figure 2:**
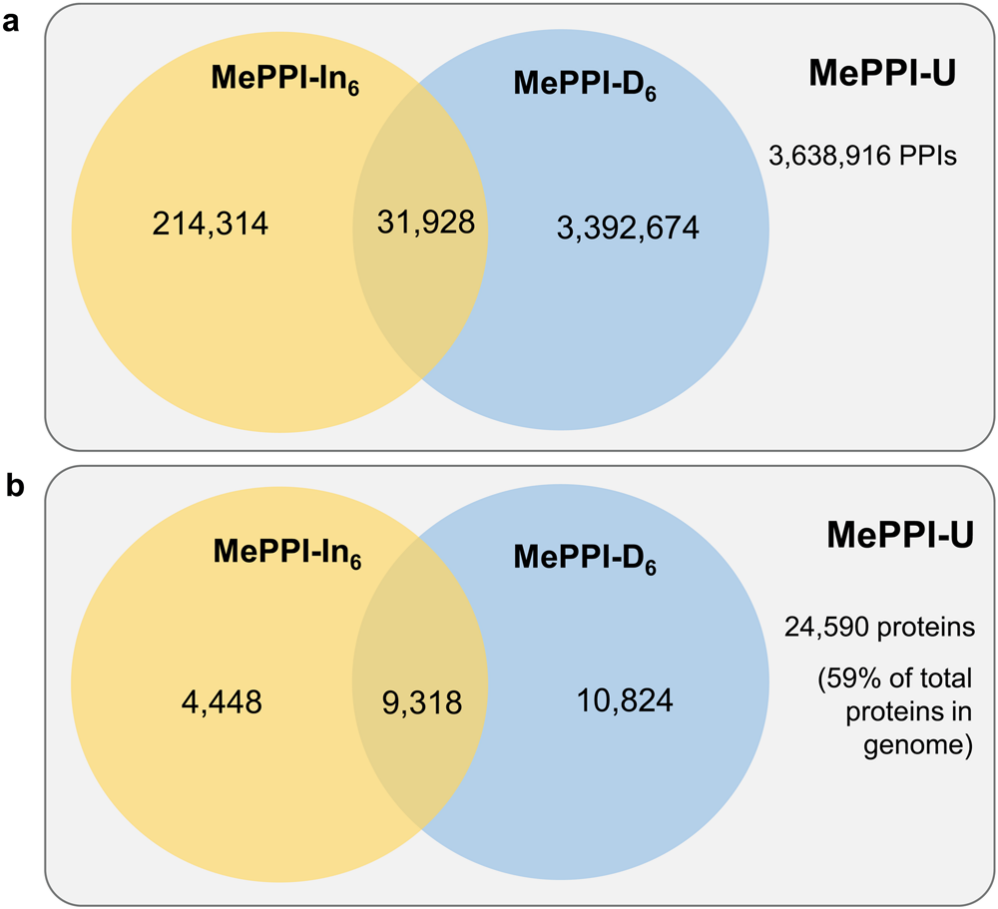
Comparison of PPI prediction from interolog-based (MePPI-In_6_) and domain-based (MePPI-D_6_) methods. (**a**) interaction comparison and (**b**) protein comparison.

The interolog-based and domain-based PPI networks were combined to yield the PPI network that represents all possible protein-protein interactions in cassava, named MePPI-U (publicly available at http://bml.sbi.kmutt.ac.th/ppi2).The MePPI-U contained 3,638,916 putative interactions and 24,590 proteins (59 percent of proteins in the cassava genome) (Fig. S1). Of the overall putative PPIs included in MePPI-U, 214,314 PPIs were derived only by the interolog-based method, 3,392,674 PPIs were only by the DDI-based method, and 31,928 PPIs were by both methods (0.9 percent of total prediction) (Fig. 2). With a total of 3,638,916 putative PPIs, the protein coverage of MePPI-U increased by 26 and 10 percent when compared with predictions by the interolog-based and domain-based method, respectively. Since the interolog- and domain-based methods utilize different principles to predict PPIs, the combined results not only allow us to compare the information from both sides, but also to minimize limitations of each individual method. The small overlap indicated different groups of PPIs (proteins) predicted from the two methods; thus, using both methods could predict interactions in broader groups of proteins (Fig. 2b). The MePPI-U network is thus proposed as a large-scale interactome network of cassava that describes broad classes of PPIs, including physical interactions between domains.

In comparison, MePPI-U contains more unique putative PPIs (3,607,054) than MePPI-In_4_^56^ (58,311) with an overlap of 31,862 putative interactions (Fig. S3). The 58,311 putative interactions in MePPI-In_4_^56^ were not included in MePPI-U because of the substantial changes in the cassava genome sequence and PPI information in literature. Of the unique 58,311 PPIs in MePPI-In_4_^56^, 12,478 PPIs were lost during the genome sequence improvement (Fig. S2b,c). Sequence re-annotation affects the homology-based analysis; updating the cassava genome from cassava V.4 to V.6 caused a mismatch of some template proteins with their counterparts in cassava, resulting in the loss of at least 40,215 putative PPIs (see an example in Fig. S4). In addition, the genome update resulted in changes in some protein sequences relative to the previous version. For example, the sequence of limit dextrinase (LD) protein (Manes.10G051700.1.p) in cassava V.6 was used to represent two proteins (cassava4.1_024672m and cassava4.1_004771m) in cassava V.4. Thus, the PPIs related to cassava4.1_024672m and cassava4.1_004771m could not be found in MePPI-U. Table 1 summarizes the comparison of MePPI-U and the previously published PPI network of cassava, MePPI-In_4_^56^. Since MePPI-U was developed from the more updated information, it covered up to 59 percent of proteins in the current cassava genome, in contrast to MePPI-In_4_^56^, which covered 21 percent of proteins in cassava genome V.4.

**Table 1:** Comparison of cassava PPI networks between previous work^56^ and MePPI-U.

Descriptions	MePPI from our previous work^56^	MePPI-U
Approaches	Interolog-based method	Interolog- and domain-based methods
Data sources	Cassava genome V.4	Cassava genome V.6
	7 plant templates	9 plant templates
	DDI information V.1	DDI information V.2
Number of proteins in genome	34,151	41,381
Number of proteins in PPI network	7,209	24,590
Number of PPIs	90,173	3,638,916
Percent proteins coverage in genome	21	59

### Validation of MePPI-U with expression data and yeast two-hybrid study

**Figure 3:**
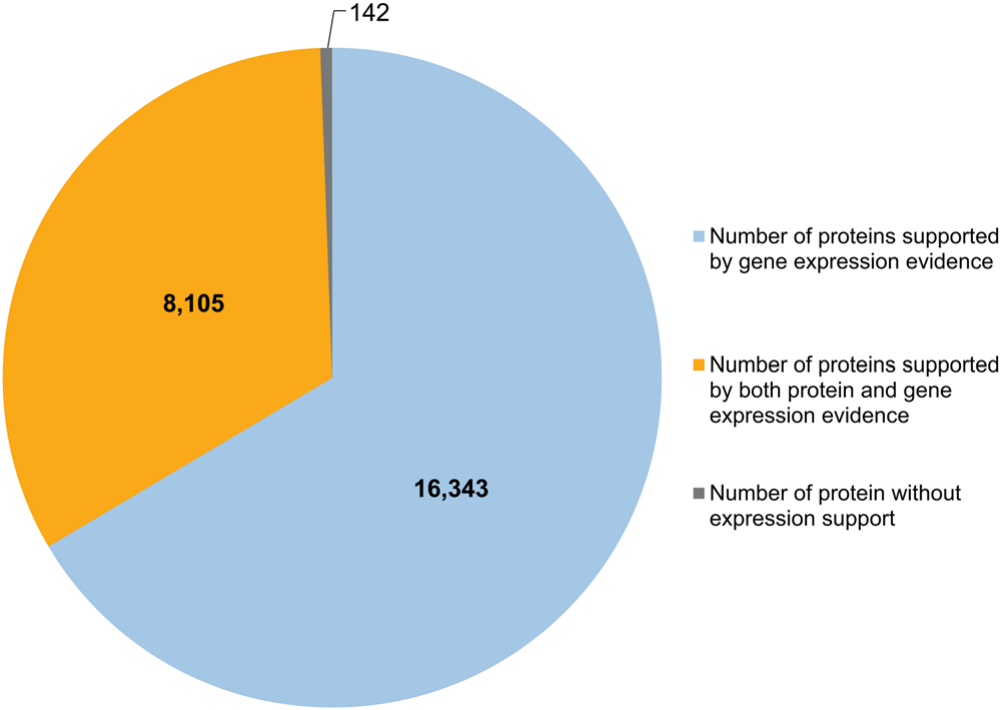
The coverage of proteins in MePPI-U supported by expression data. All 24,590 proteins in MePPI-U were matched with published cassava protein and/or gene expression data^49–54,62–70^ to support their existence in MePPI-U.

The putative PPIs in the MePPI-U network were consolidated with the transcriptome and proteome expression data. First, the expression of genes or proteins was employed to confirm the presence of those proteins in the network, by assuming that the interactions of proteins could only be achieved when proteins or the corresponding protein-coding genes are expressed. Thus, the expression information was employed to validate the prediction of PPIs in MePPI-U. The presence of proteins in MePPI-U was verified using fifteen collective expression datasets of expressed genes and protein expression data, which consisted of seven set of protein and eight set of gene expression (see Methods). The expression data supported 99 percent of proteins (24,448 proteins) in MePPI-U; 8,105 proteins (~ 33 percent of proteins in MePPI-U) were supported by both the protein and gene expression data (Fig. 3). The confirmed proteins thereby supported the occurrence of 3,612,250 interactions in the MePPI-U network. Second, the predicted interactions were validated further with the co-expression profile of paired proteins, by assuming that the co-expressed proteins or genes have a higher probability of interacting. For each predicted PPI, Pearson correlation analysis was performed to examine the concurrent expression of a protein pair in order to support the interaction. The five time-series datasets of Naconsie *et al*.^49^, Li *et al*.^52^, Amuge *et al*.^69^, An *et al*.^63^ and Wang *et al*.^70^ used in this study could support 4,742 interactions in MePPI-U (Table 2). The remaining PPIs in the network would have to be validated when more applicable time-series expression datasets become available.

**Table 2:** Validation of predicted PPIs in MePPI-U through the consistence of cassava gene/protein expression profiles.

Datasets	Conditions*	Organs	Cultivars	Platforms	Number of genes/ proteins	PPIs supported by co-expression**
**Gene level**						
Amuge *et al*. (2017)^69^	CBSV infection (0 hr, 6 hr, 24 hr, 48 hr, 5 dag, 8 dag,45 dag and 54 dag)	leaf	Namikonga and Albert	RNA-seq	33,033	4,595
Wang *et al*.(2014)^70^	root development (75, 120 and 150 dap)	storage root	KU50, Arg7	RNA-seq	30,666	1,111
An *et al*. (2012)^63^	cold stress (7 °C at 0, 4 and 9 hr)	apical shoot	TMS60444	microarray	20,840	3
Li *et al*. (2010)^64^	development (60, 120, 210 and 300 dap)	leaf, stem, root		microarray	2,878	6
**Protein level**						
Naconsie *et al*.(2016)^49^	root development (3, 6, 9 and 12 month)	storage root	KU50	2D-gel	67	6
						4,742

^*^ hr = hour, dag = days after grafting and dap = days after planting.

** Criteria: RNA-seq platform: expression ≥ 80 percentile, *PCC* > 0.99 and p-value <0.1.

Microarray platform: expression ≥ 80 percentile, *PCC* > 0.90 and p-value <0.1.

**Figure 4:**
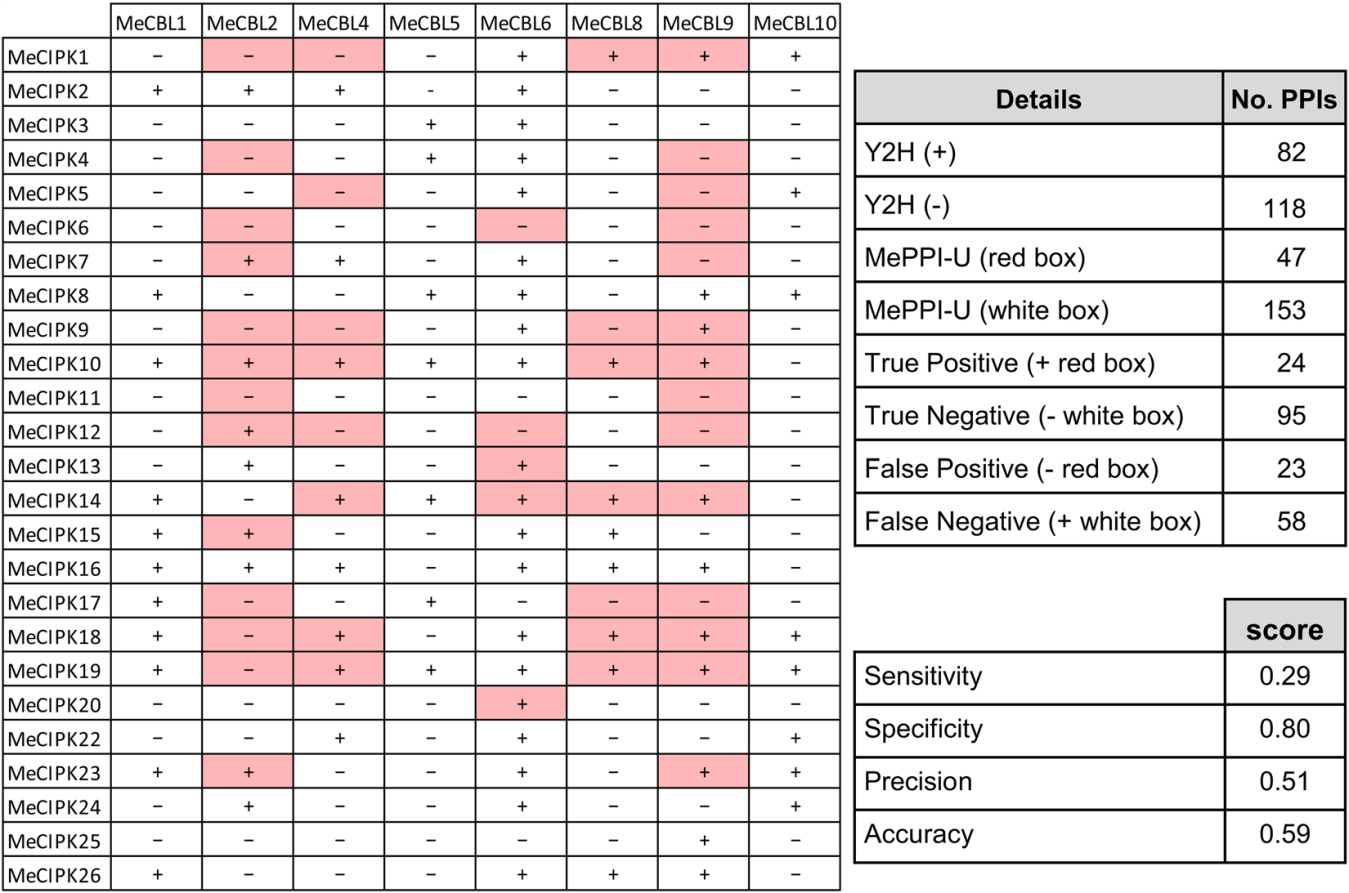
Interactions of MeCBL and MeCIPK proteins by prediction and yeast two-hybrid (Y2H) system. The box color represents the results from prediction: red (interaction) and white (no interaction). The symbols represent the results from Y2H; + (interaction) and − (no interaction) from Y2H.

Furthermore, MePPI-U was partially validated using PPI data from the yeast two-hybrid method. The 200 interactions between eight CBLs and 25 CIPKs proteins from MePPI-U were compared to yeast two-hybrid results^71^ (Fig. 4). The confusion matrix showed that 119 predicted interactions were supported by yeast two-hybrid, with 24 true positives and 95 true negatives. On the other hand, 81 predicted interactions could not match with Y2H data, with 23 false positives and 58 false negatives. Our predictions, in MePPI-U, showed high accuracy (0.6) and specificity (0.8) notwithstanding the size limitation of data used for the validation, which indicates its reliability.

### Confidence scoring of MePPI-U

The predicted PPIs in MePPI-U were assigned a confidence value ($CV$) score to represent the amount of collective information underlying the prediction (see Methods). The $CV$ score ranges from 0 to 1, indicating low to high levels of confidence. Figure S6 showed that the majority of predicted PPIs in MePPI-U had a low $CV$ score, as shown by the positively skewed distribution in which only 95,203 PPIs had a $\,CV\,$> 0.5 (2.6 percent of total predicted PPIs). This was because most of the interolog-based PPI predictions were computational from template species rather than by experimental measurements, and those by the domain-based method were mostly from proteins with a single domain. Accordingly, the factor $M$ for the calculation of $C{V}_{interolog}$ and the correction factor *d* for the calculation of $C{V}_{DDI}$ were often 0.5, therefore, the highest $\,CV$ score in this network was 0.5. This $CV$ score should help contrast the reliability of each prediction based on collective support evidence. The predictions and $CV\,$value could be improved in the future as more data become available.

### Topology and functional content of MePPI-U

The MePPI-U showed scale-free properties as demonstrated by the connectivity ($k$) of the proteins in the network, which followed a power-law distribution, $P(k) \sim \,{k}^{-\gamma }$. The observed topology suggested biological network characteristics of MePPI-U where most of the network constituents were linked by a few hub proteins (Fig. S7). The results correspond with the previously proposed interactome network of cassava^56^, although the size and coverage of the two networks differ. Similar topological characteristics were also observed in broad interactome networks of plants, including Arabidopsis^22^, rice^23^, sweet orange^47^ and tomato^25^. In MePPI-U, the top hub proteins included heat shock proteins (HSP) such as HSP70, HSP80 and HSP90, which are found in Arabidopsis^22^ and rice^23^ PPI networks as well as in MePPI-In_4_ proposed by Thanasomboon *et al*.^56^.

**Figure 5:**
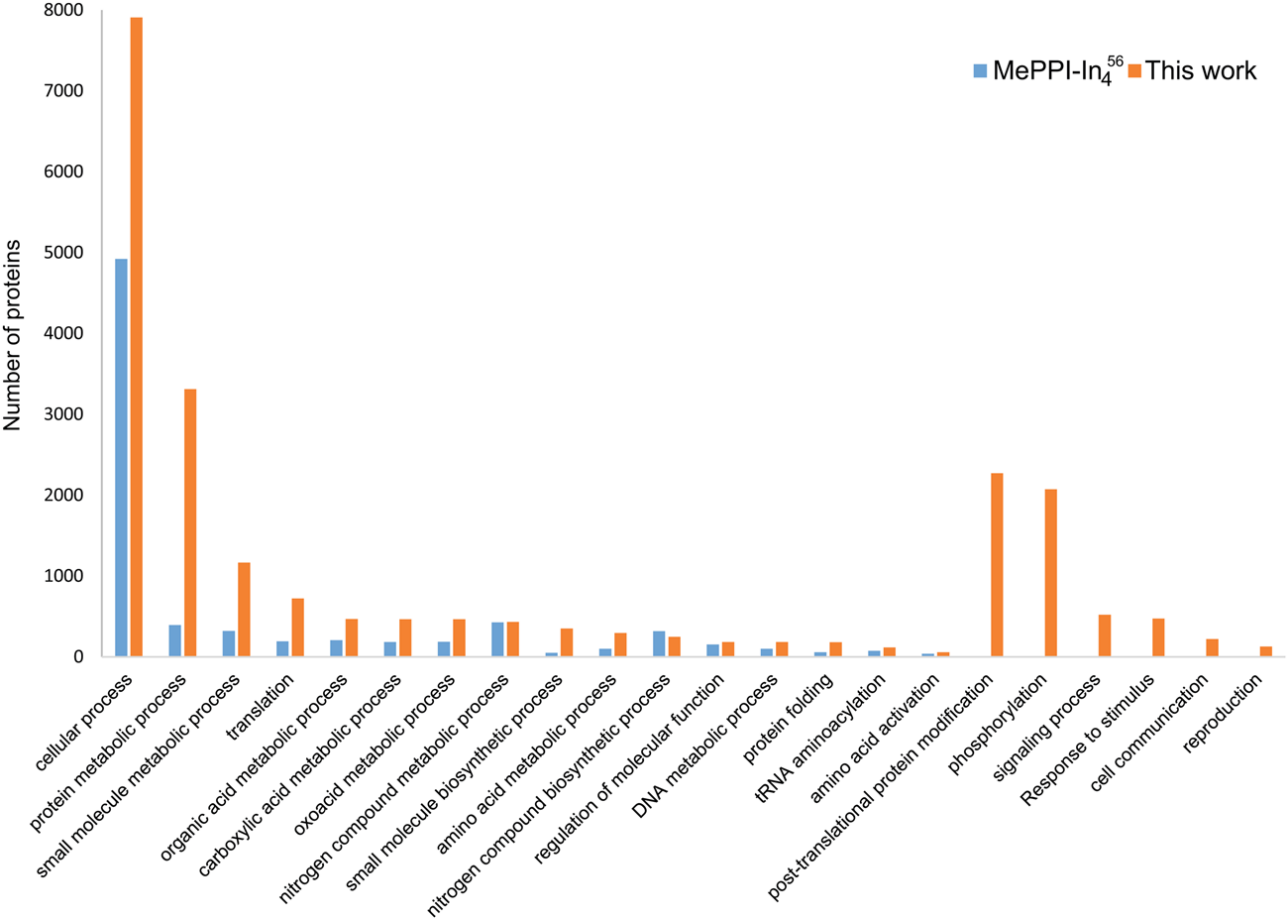
Comparison of protein functions in cassava PPI networks, MePPI-In_4_^56^ and MePPI-U, analysed based upon GO enrichment (*p*-value <0.05) in biological process class.

With a great number of proteins, MePPI-U could extensively describe proteins and PPIs involved in biological functions. GO analysis of proteins in MePPI-U, presented in Figure S8, showed that the enriched biological processes of PPIs were related to cellular protein metabolism, protein modification, post-translational modification, and protein phosphorylation. These results corresponded to the predominant molecular functions of the interacting proteins involved in protein kinase activity. A comparison of the functional properties of MePPI-U and MePPI-In_4_^56^ revealed greater numbers of biological processes covered by putative PPIs in MePPI-U, especially post-translational modification and signaling (Fig. 5).

### Integrative interactome network and the inference of dynamic interaction unwiring and rewiring PPI networks

**Figure 6:**
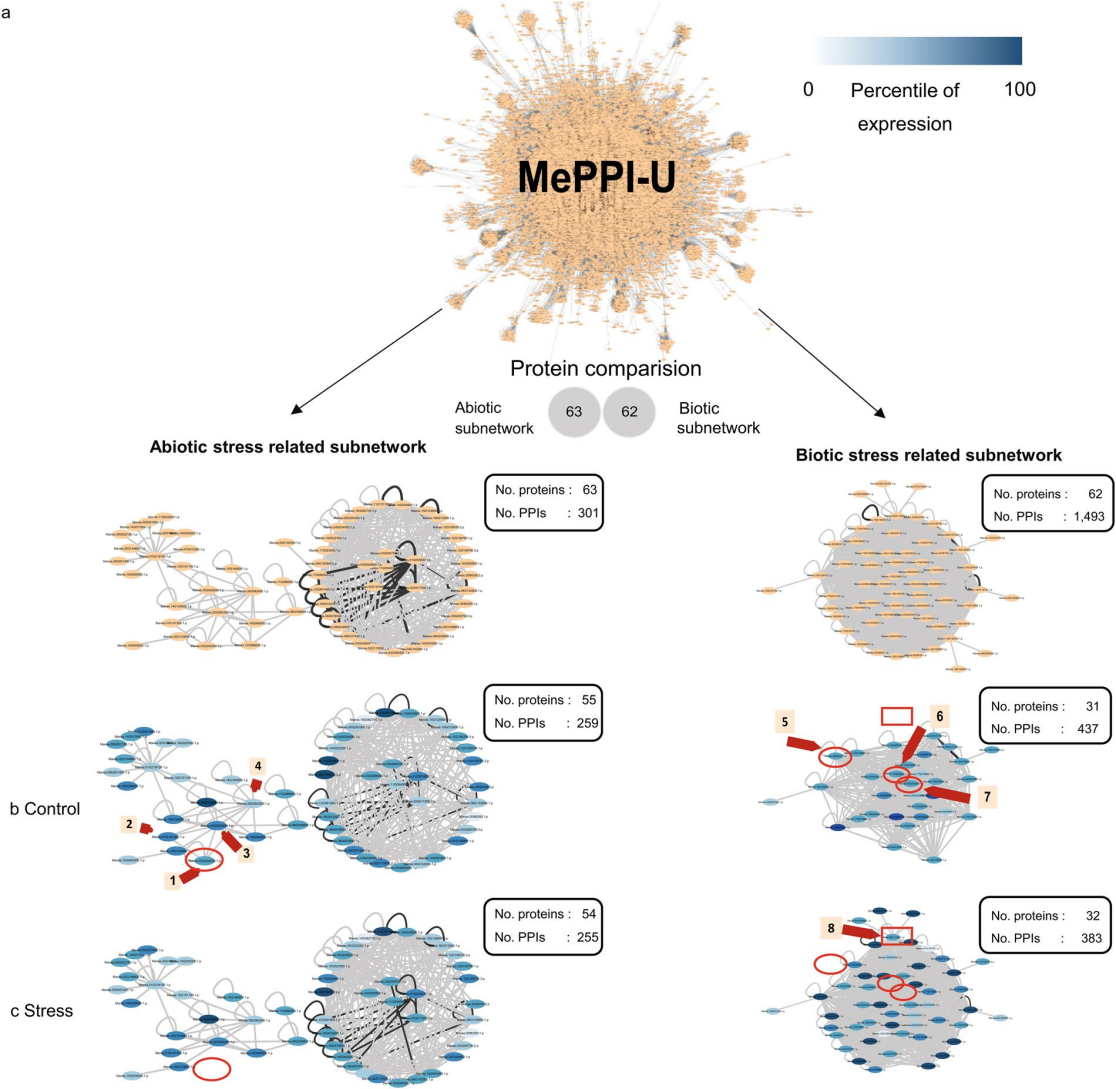
Dynamic MePPI-U for different conditions: (**a**) abiotic and biotic stress-related subnetworks inferred from MePPI-U using GO information. Both subnetworks were integrated with expression data under CBSV infection (**b**) and drought stress (**c**). The nodes and edges represent proteins and their interactions, repectively. The node colors show proteins from abotic and biotic stress related subnetwork (orange) and percentile expression of proteins from highest (dark blue) to lowest (light blue) while the edge colors represent PPIs from both interolog- and domain- based prediction (black) or either interolog or DDI based method (grey). The circles and rectangles marked proteins that showed different interactions in MePPI-U of control and stress conditions. The number in orange boxes denote the following proteins; (1) adenine nucleotide alpha hydrolases-like superfamily protein (ANAH; Manes.03G204200.1.p), (2) thioredoxin H-type1 (TRXH1; Manes.01G141300.1.p), (3) hypoxia responsive universal stress protein 1 (HRU1; Manes.02G080300.1.p), (4) universal stress protein (USP; Manes.08G082400.1.p), (5) major latex protein-like protein 28 (MLP28; Manes.S038200.1.p), (6–7) major latex protein-like protein 423 (MLP423; Manes.03G200500.1.p and Manes.15G008000.1.p) and 8) abscisic acid receptor PYL12 (PYL12; Manes.03G115300.1.p).

In living organisms, PPIs might occur only when the function of interacting proteins is needed^76^. While some PPIs stay intact till being triggered, many PPIs occur shortly and also change over time and environments^77^. The network of protein interaction is thus dynamically evolved by unwiring and rewiring of the interacting proteins, known as a dynamic PPI network (DPIN)^78^. A DPIN could be inferred from a reference interactome network by incorporating gene expression data, as information of state^36,79^. The integrative interactome network presumably represents the timely protein-protein interaction by assuming that only expressed proteins could interact. The integrative MePPI-U network (MePPI-Ux) was constructed to investigate the DPIN of cassava. Figure 6 shows examples of MePPI-Ux constructed under biotic and abiotic stress conditions, based on the biotic and abiotic GO terms of proteins (GO:0009607 and GO:0009628 respectively). The biotic stress-related subnetwork contained 62 proteins (GO:0009607) with 1,493 inferred interactions, whereas the abiotic stress-related subnetwork contained 63 proteins (GO:0009628) with 301 inferred interactions (Fig. 6a). Thus, both subnetworks contained a similar number of proteins, but the constituent members and number of interactions differed. The DPINs of cassava under biotic and abiotic stresses were subsequently constructed by incorporating transcriptome data measured under cassava brown streak virus (CBSV) infection^69^ and drought stress^68^, denoted as MePPI-Ux_CBSV_ and MePPI-Ux_drought_, respectively.

The inferred PPIs (iPPIs) in the biotic stress-related subnetwork differed from those in the normal condition (Fig. 6b,c, right panel). The integrative interactome subnetwork under CBSV infection (MePPI-Ux_CBSV_) consisted of 383 iPPIs and 32 proteins (Fig. 6c, right panel). The major latex protein-like protein 423 (MLP423; Manes.03G200500.1.p and Manes.15G008000.1.p) and major latex protein-like protein 28 (MLP28; Manes.S038200.1.p)) were absent under CBSV stress conditions (Fig. 6b,c, right panel, red circle), resulting in the loss of 78 edges among MPL proteins in the network, such as MLP31, MLP43 and MLP328. The presence of abscisic acid receptor PYL12 (PYL12; Manes.03G115300.1.p) (Fig. 6b,c, right panel, red rectangle) introduced 57 more interactions from the normal condition, allowing linkages between PYL12, MPL and a serine/threonine phosphatase 2CHAB1 (HAB1; a serine/threonine phosphatase) to form. The MPL and HAB1 proteins were reported to be involved in the defense mechanisms of plants^80,81^, and in the dephosphorylation process that regulate cellular stress responses in eukaryotes^82^, respectively. Also, MPL and PYL proteins contain the Bet v1 domain in their sequences and are known to be involved in the defense process of Panax ginseng^81^ and birch pollen^83^. The findings corroborate a previous study that showed the knockdown of MPL-like protein expression resulted in increased susceptibility of cotton plants to *Verticillium dahliaei* infection^80^. Therefore, changes in protein interactions might be associated with the response of plants to infections.

The integrative interactome subnetwork under drought stress (MePPI-Ux_drought_) composed of 255 iPPIs and 54 proteins (Fig. 6c, left panel). Absence of the adenine nucleotide alpha hydrolases-like superfamily protein (ANAH; Manes.03G204200.1.p) (Fig. 6b,c, left panel, red circle) impaired 4 edges, one of which represented self-interaction and the other three linked ANAH with thioredoxin H-type1 (TRXH1; Manes.01G141300.1.p), hypoxia responsive universal stress protein 1 (HRU1; Manes.02G080300.1.p) and universal stress protein (USP; Manes.08G082400.1.p), which have been linked to stress response in *Sorghum bicolor*(L.) Moench^84^. ANAH was identified as a putative stress responsive gene based on *cis* regulatory elements^85^, and the protein it encodes interacts with several stress response proteins^86,87^. Accordingly, we hypothesized that ANAH may play role in connecting stress response proteins to makes them synchronously function under this particular condition. Relative to the normal condition, changes in MePPI-Ux_drought_ was subtler than in MePPI-Ux_CBSV_ subnetworks (Fig. 6b,c), which might indicate differences in the PPI-related regulatory process in response to the stress.

### Insights into dynamic PPI network (DPIN) of starch biosynthesis through the integrative interactome network

Cassava is always valued based upon the yield of storage roots as well as starch content. However, these characters often vary according to changes in environmental conditions^63,88,89^, irrespective of the genetic similarity. An *et al*. (2013) showed the structure of chloroplasts in cassava leaves was affected by cold stress, which resulted in decreased thylakoid number and organization and loss of starch granules^63^. However, the total sugar content remained unchanged when compared to the control condition^63^. Based on these findings, it was indicated that cassava, a tropical crop adapted to warm climate, has cold responsive genes similar to temperate plants (e.g. Arabidopsis). The results indicated that the ability of cassava to tolerate cold might not only be due to the amount of cold responsive genes, but might also involve other regulatory systems^63^. Here, the integrative interactome network was employed to study the influence of cold stress on the starch production process in cassava, with respect to changes in iPPIs related to starch biosynthesis.

**Figure 7:**
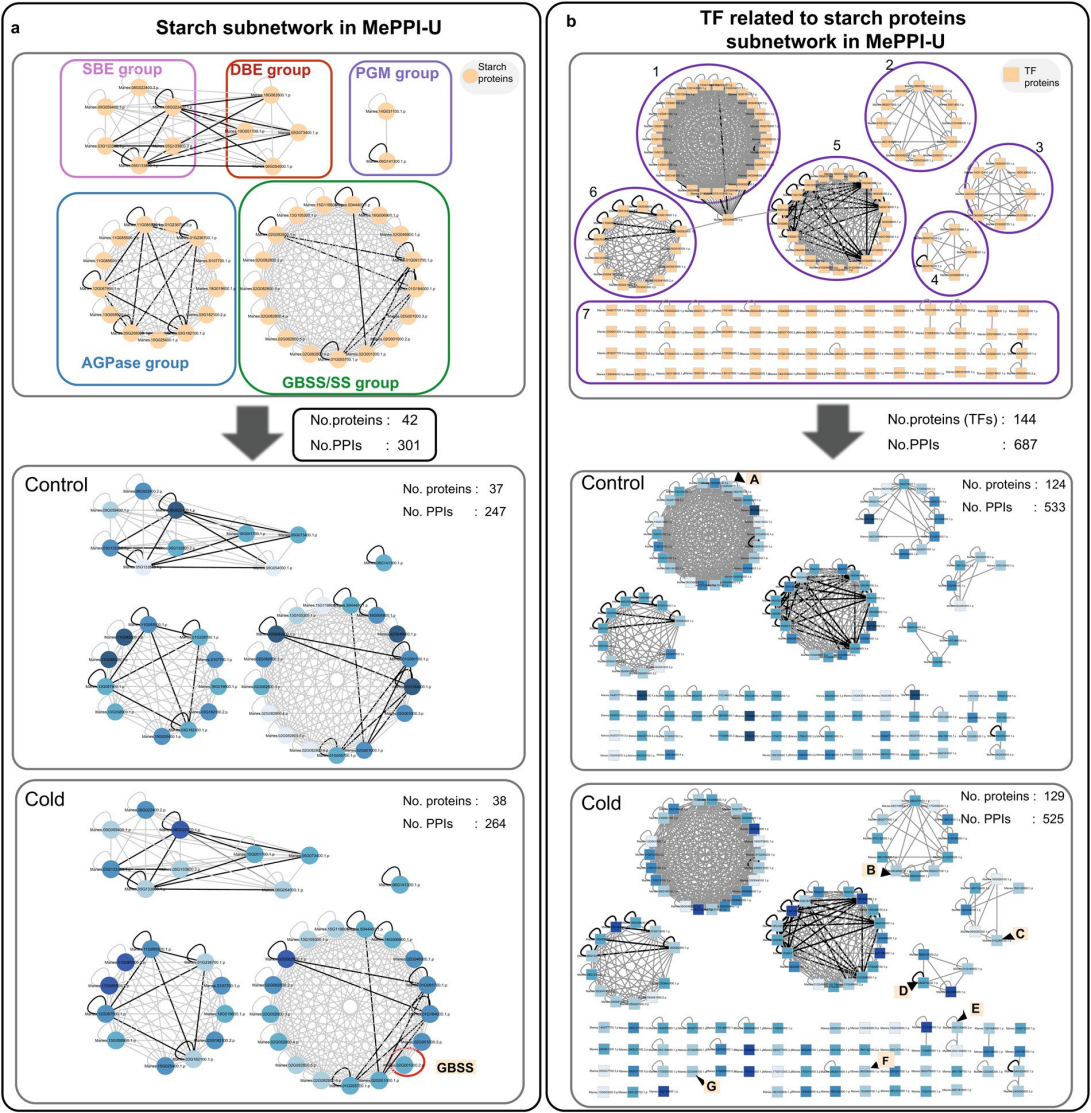
Dynamic starch and TF subnetworks in cold stressed condition. (**a**) starch subnetwork was classified into five functional groups; (1) phosphoglucomutase (PGM), (2) glucose-1-phosphate adenylyltransferase (AGPase), (3) starch synthase (SS), (4) 1,4 –alpha-glucan branching enzyme (SBE) and (5) 1,4 –alpha-glucan debranching enzyme (DBE). The red circle represents Manes.02G001000.2.p (GBSS2) protein that showed different interactions relative to control. (**b**) TFs related to the starch protein subnetwork were separated in 7 groups; (1) ethylene response factor proteins (ERF), (2) Myb domain proteins (MYB), (3) basic helix-loop-helix (bHLH) DNA-binding proteins, (4) NAC domain containing proteins, (5) basic-leucine zipper (bZIP) transcription factor proteins (6) homeobox proteins and (7) other TF proteins. The nodes represent starch (circle) and transcription factor (square) proteins, while the edges show the interactions. The node colors show percentile expression of proteins from highest (dark blue) to lowest (light blue), while the edge colors represent PPIs from both interolog and DDI based predictions (black), or either (grey). The alphabet in orange boxes denote the following TFs proteins; (**A**) ERF transcription factor protein (ERF; Manes.16G034200.1.p), (**B**) Myb domain protein 30 (Myb30;Manes.02G046100.1.p), (**C**) bHLH DNA binding protein (bHLH; Manes.01G269700.1.p), (**D**) NAC containing protein (NAC;Manes.06G015000.1.p), (**E**) NIN-like protein 5 (NIN5; Manes.05G130800.2.p), (**F**) OBF binding protein 4 (OBF4; Manes.06G080600.1.p) and Myb domain protein 96 (Myb96; Manes.06G092600.1.p).

A total of 42 proteins were identified for the starch biosynthesis metabolic pathway. These proteins were classified into five groups: (1) phosphoglucomutase (PGM), (2) glucose-1-phosphate adenylyltransferase (AGPase), (3) starch synthase (SS), (4) 1,4 –alpha-glucan branching enzyme (SBE) and (5) 1,4 –alpha-glucan debranching enzyme (DBE). Within MePPI-U, 301 putative interactions of these 42 proteins were identified. (Fig. 7a, top panel). This suggests that metabolic proteins involved in the starch biosynthesis pathway well interact with each other, but mostly within the same group of functional proteins. Interactions of PGM, SS and AGPase proteins were all found within their own group, whereas interactions linking the two enzymatic groups were only found among SBE and DBE proteins. These results implied a close connection between SBE and DBE proteins while functioning in the starch biosynthesis process. As reported in amylopectin synthesis^90^, these interactions might be required to synthesize starch with precise molecular structure, which is crucial for starch granule formation.

The integrative interactome subnetwork of starch biosynthesis under cold stress was constructed by incorporating expression data^68^ into the MePPI-U network (Fig. 7a). Compared to the normal condition, interactions among cassava starch proteins seemed to change slightly once exposed to cold.The appearance of granule bound starch synthase 2 (GBSS2, Manes.02G001000.2.p) would introduce 17 more interactions (Fig. 7a, red circle). Additionally, we hypothesized that cold stress might not affect only the interactions between starch metabolic proteins, but might have an influence on how each of the starch genes might be regulated. To observed this, the integrative interactome subnetwork of starch protein regulators, i.e. transcription factors (TFs), was performed (Fig. 7b). According to PlantTFDB database^91^, 144 transcription factors of the 42 starch proteins were inferred based on *cis*-regulatory element analysis. They included 7 major families, (1) ethylene response factor protein (ERF), (2) Myb domain protein (MYB), (3) basic helix-loop-helix (bHLH) DNA-binding protein, (4) NAC domain containing protein, (5) basic-leucine zipper (bZIP) transcription factor protein (6) homeobox protein and (7) WRKY DNA-binding protein. In total, 687 putative PPIs among 144 TFs were found in MePPI-U (Fig. 7b). Under cold stress, changes in expression of seven TFs were observed. These included bHLH DNA binding protein (bHLH; Manes.01G269700.1.p), ERF transcription factor protein (ERF; Manes.16G034200.1.p), Myb domain protein 30 (Myb30;Manes.02G046100.1.p), Myb domain protein 96 (Myb96; Manes.06G092600.1.p), NAC containing protein (NAC;Manes.06G015000.1.p), NIN-like protein 5 (NIN5; Manes.05G130800.2.p) and OBF binding protein 4 (OBF4; Manes.06G080600.1.p). According to PlantTFDB, these TFs are involved in governing the transcription of AGPase, SS and ISA genes, i.e., bHLH controlling GBSS1, ERF controlling APS1-2, Myb30 controlling ISA3 and APS1-1, Myb96 controlling ISA3, NAC controlling APS1-1 and APS1-2, NIN5 controlling APL1-1 and SS3, and OBF4 controlling APL2-2 and SS2-1 (Table S2). The results suggested that cold stress might affect starch biosynthesis through changes in PPI-mediated regulation at the transcriptional regulatory level, especially when related to rate-limiting enzymatic proteins such as AGPase.

**Figure 8:**
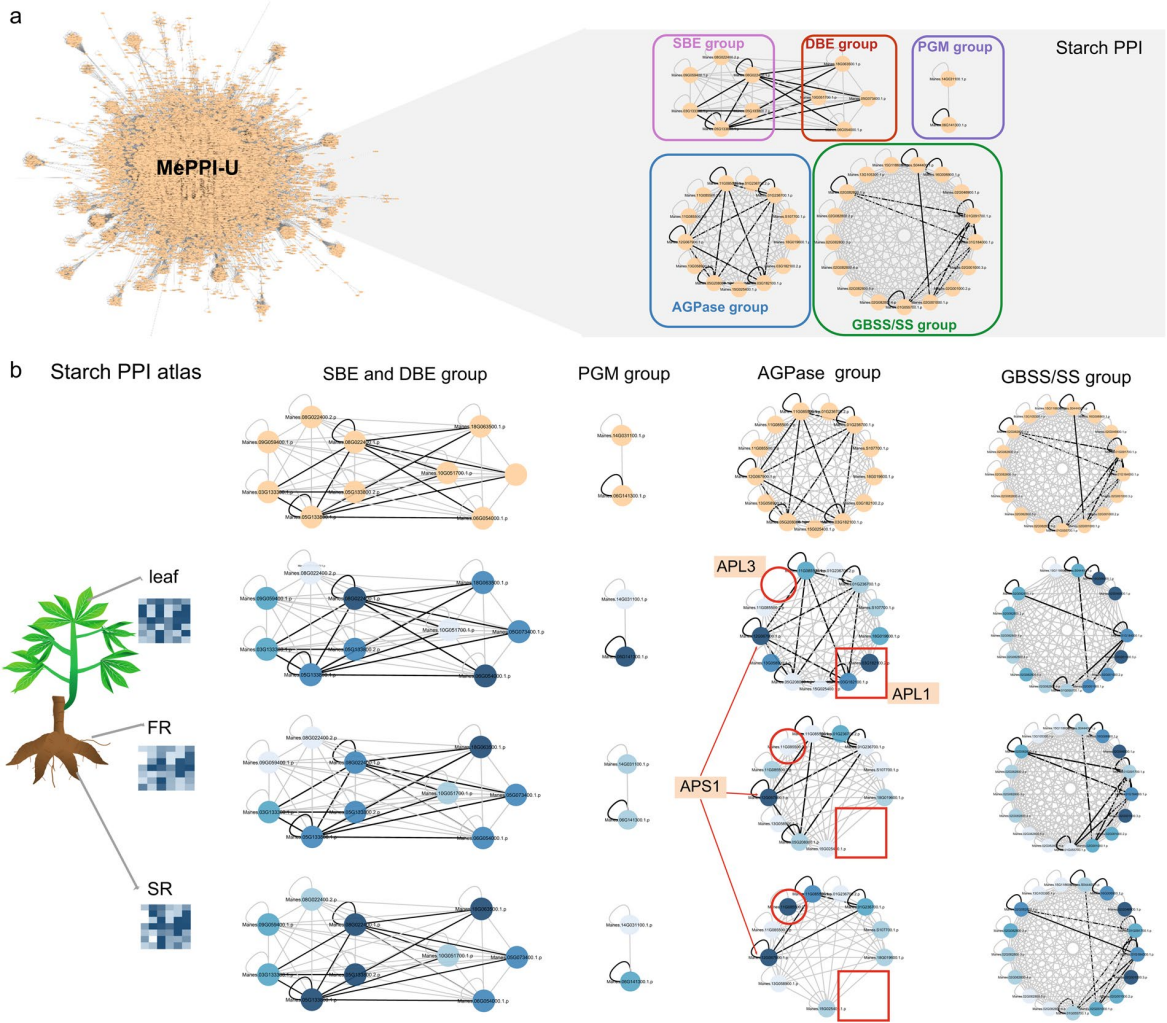
Starch PPI in leaves, fibrous roots and storage roots: (**a**) the starch subnetwork from MePPI-U and (**b**) the dynamic starch subnetwork in leaves, fibrous roots (FR) and storage roots (SR). The nodes and edges represent proteins and their interactions, repectively. The node colors show percentile expression of proteins from highest (dark blue) to lowest (light blue), while the edge colors represent PPIs from both interolog and DDI predictions (black), or either (grey).

Besides investigating the DPIN underlying starch biosynthesis during cold stress condition, a similar study was performed to gain more insights into the interactome network of starch proteins in leaf and root tissues. Although starch is synthesized in both tissue types, the aim of the process is different. In leaves, starch is formed to allocate sugars obtained from the photosynthesis process, whereas starch in roots is synthesized for storage purpose. Different interactions of starch proteins in these tissues were thus inferred, and the results are presented in Fig. 8. Integrative interactome networks of starch proteins in leaf (MePPI-Ux_SB,leaf_) and root (MePPI-Ux_SB,root_) tissues suggest a difference in AGPase protein interaction (Fig. 8b). AGPase is a complex heterotetrameric enzyme with 2 small and 2 large protein subunits required for starch biosynthesis. The expression of the small AGPase subunits (APS) in leaves and roots was comparable (with less than a two-fold change of percentile rank between both tissues), while the difference in the expression of the large subunits (APL) in both tissues was substantial (Fig. 8). These results corresponded to previous reports that the small subunits are primarily catalytic, while the large subunits are mainly regulatory^92–94^. The difference in APL genes expression may thus lead to distinct interactions that may be specific to each tissue type (Fig. 8, red circle and rectangle). Taken together, the integrative interactome network enabled us to envisage the changes in iPPIs underlying the phenotype under different conditions. The invloved PPIs inferred under specific conditions would provide useful information to identify protein complexes that would give us a clue of possible regulatory mechanism. Tang *et al*.(2011)^79^ showed that the protein complexes predicted from DPINs are more functionally coherent than those derived from a static PPI network. Moreover, DPIN is exploited to find the dynamic network biomarkers which can also be monitored at different stages and time points during the development of diseases^95^.

## Conclusions

The interactome network of cassava was constructed by interolog-based and domain-based approaches to improve coverage of PPIs at the genome-wide level. The resulting network, named MePPI-U, contains 3,638,916 putative PPIs interconnecting 24,590 proteins, which represents 59 percent of entire proteins in cassava, a 38 percent increase from the previous network (MePPI-In_4_^56^). Expression data were integrated into the MePPI-U to yield MePPI-Ux that suggests iPPIs under specific conditions. The MePPI-Ux was used to investigate condition-dependent PPIs under drought stress, cassava brown streak virus (CBSV) infection, and starch biosynthesis in leaf and root tissues. MePPI-Ux_CBSV_ and MePPI-Ux_drought_ suggest iPPIs that could be involved in cassava response to stress. Moreover, MePPI-Ux_SB,leaf_ and MePPI-Ux_SB,root_ suggest the different interactions of enzymatic proteins between tissues may be modulated by interactions of their TF proteins. This integrative MePPI-U network with expression data leads to more insights into PPI-related regulation that would help cassava starch improvement in both quality and quantity.

## Supplementary information

Supplementary Information.
