# Supplementary material for: Exploring dynamic protein-protein interactions in cassava through the integrative interactome network
Source: Sci Rep. 2020 Apr 16;10:6510. doi: 10.1038/s41598-020-63536-0 (PMC7162878; doi:10.1038/s41598-020-63536-0)
Supplement: Supplementary file 1 — Supplementary Information. [file 41598_2020_63536_MOESM1_ESM.pdf]

Exploring dynamic protein-protein interactions in cassava through the integrative interactome network

Ratana Thanasomboon[a], Saowalak Kalapanulak[b,c], Supatcharee Netrphan[d] and Treenut Saithong[b,c,*]

[a] Biological Engineering Program, Faculty of Engineering, King Mongkut's University of Technology Thonburi, Bangkok, 10140, Thailand
[b] Systems Biology and Bioinformatics Research Group, Pilot Plant Development and Training Institute, King Mongkut's University of Technology Thonburi (Bang Khun Thian), Bangkok, 10150, Thailand
[c] Bioinformatics and Systems Biology Program, School of Bioresources and Technology, King Mongkut's University of Technology Thonburi (Bang Khun Thian), Bangkok, 10150, Thailand
[d] National Center for Genetic Engineering and Biotechnology, Pathum Thani, 12120, Thailand

* Correspondence: treenut.sai@kmutt.ac.th

Table S1 Prediction of protein-protein interactions in cassava (MePPI-In_6) from known PPIs in plant templates

Plant	Genome information		PPI Information			MePPI-In_6	
	Number of genes	Number of proteins	Number of PPIs	Number of proteins	Sources	Number of predicted PPIs	Number of proteins corresponding to the predicted PPIs
Arabidopsis	27,416	35,386	235,215	17,962	ref.21,22, 43,45,58,59	107,235	10,046
Rice	52,424	42,190	76,829	5,219	ref. 23, 58	17,697	2,089
Maize	32,540	63,540	2,762,560	14,000	ref. 24, 58	32,894	2,642
Potato	35,119	51,472	52	66	ref.58	17	11
Poplar	41,335	73,013	8	10	ref.58	5	7
Castor bean	25,878	31,221	10	10	ref.58	1	1
Soybean	56,044	88,647	10	12	ref.58	7	7
Tomato	21,121	34,727	357,946	10,585	ref.25	97,885	5,596
Sweet orange	29,445	44,275	13,852	2,025	ref.47	2,647	908
Cassava	33,033	41,381				246,242	13,766

Table S2 List of differentially expressed transcription factors and their target genes under cold stress conditions

Differentially expressed TF proteins			Starch target genes		
Protein ID	Description	Symbol	Gene ID	Description	Symbol
Manes.06G092600.1.p	Myb domain protein 96	Myb96	Manes.18G063500	isoamylase 3	ISA3
Manes.01G269700.1.p	bHLH DNA binding protein	bHLH	Manes.01G055700	granule-bound starch synthase 1	GBSS1
Manes.06G015000.1.p	NAC domain containning protein	NAC	Manes.12G067900	ADP glucose phosphorylase small subunit 1	APS1-1
			Manes.13G058900	ADP glucose phosphorylase small subunit 1	APS1-2
Manes.05G130800.2.p	NIN-like protein 5	NIN5	Manes.15G025400	ADP glucose phosphorylase large subunit 1	APL1-1
			Manes.16G006900	starch synthase 3	SS3
Manes.16G034200.1.p	ERF/AP2 transcription factor protein	ERF	Manes.13G058900	ADP glucose phosphorylase small subunit 1	APS1-2
Manes.02G046100.1.p	Myb domain protein 30	Myb30	Manes.18G063500	isoamylase 3	ISA3
			Manes.12G067900	ADP glucose phosphorylase small subunit 1	APS1-1
Manes.06G080600.1.p	OBF binding protein 4	OBF4	Manes.01G236700	ADP glucose phosphorylase large subunit 2	APL2-2
			Manes.01G091700	starch synthase 2	SS2-1

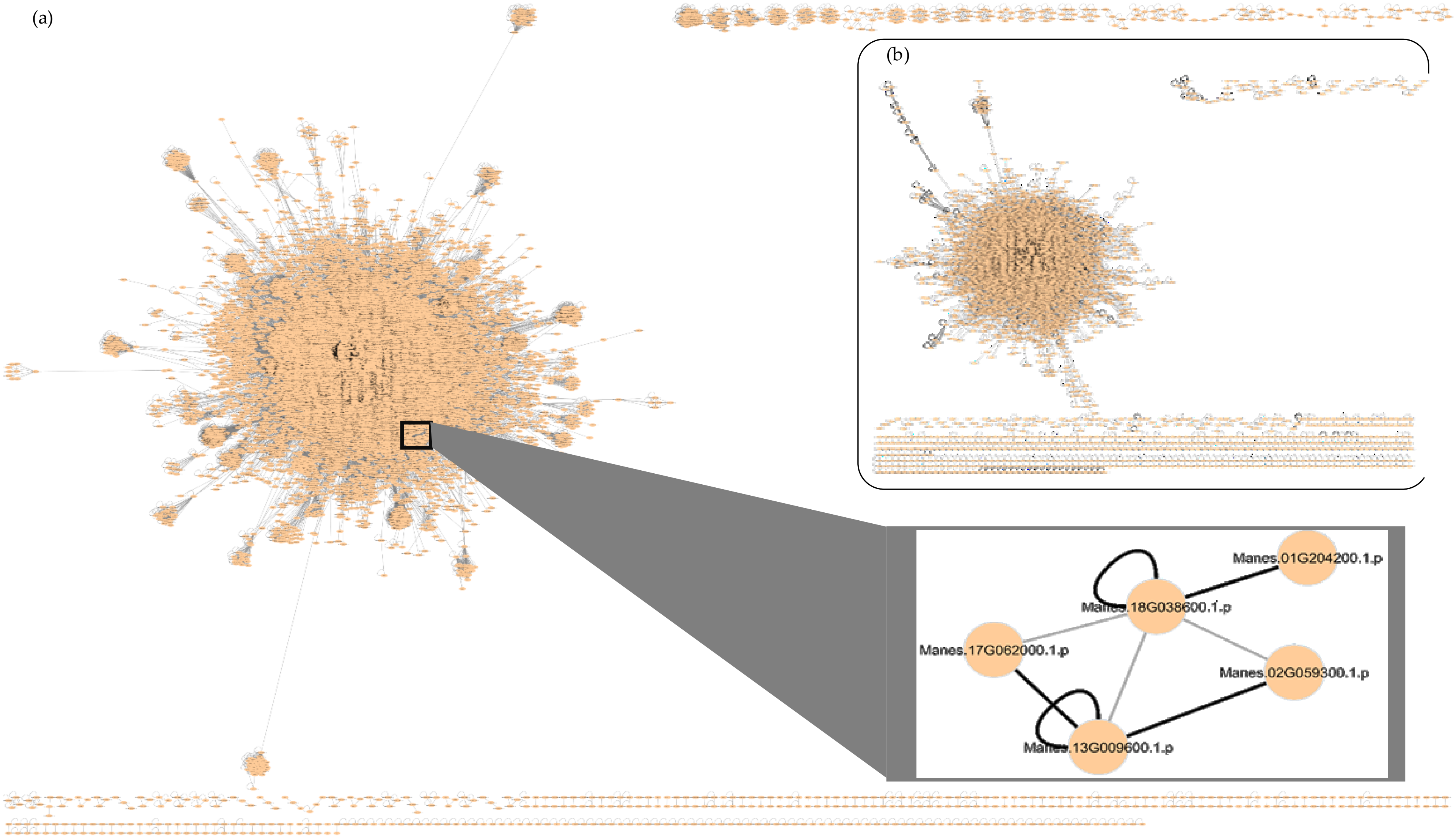

Figure S1 The constructed protein-protein interaction network of cassava. a) The MePPI-U (this work) contained 3,638,916 interactions (edges) interconnecting 24,590 proteins (nodes). b) PPI network from Thanasomboon et al (2017) [56] consisted of 90,173 interactions with 7,209 proteins. Different colors were given to edges. The black edges represent PPIs from both interolog and DDI based prediction while the grey ones represent those from either interolog- or domain- based method.

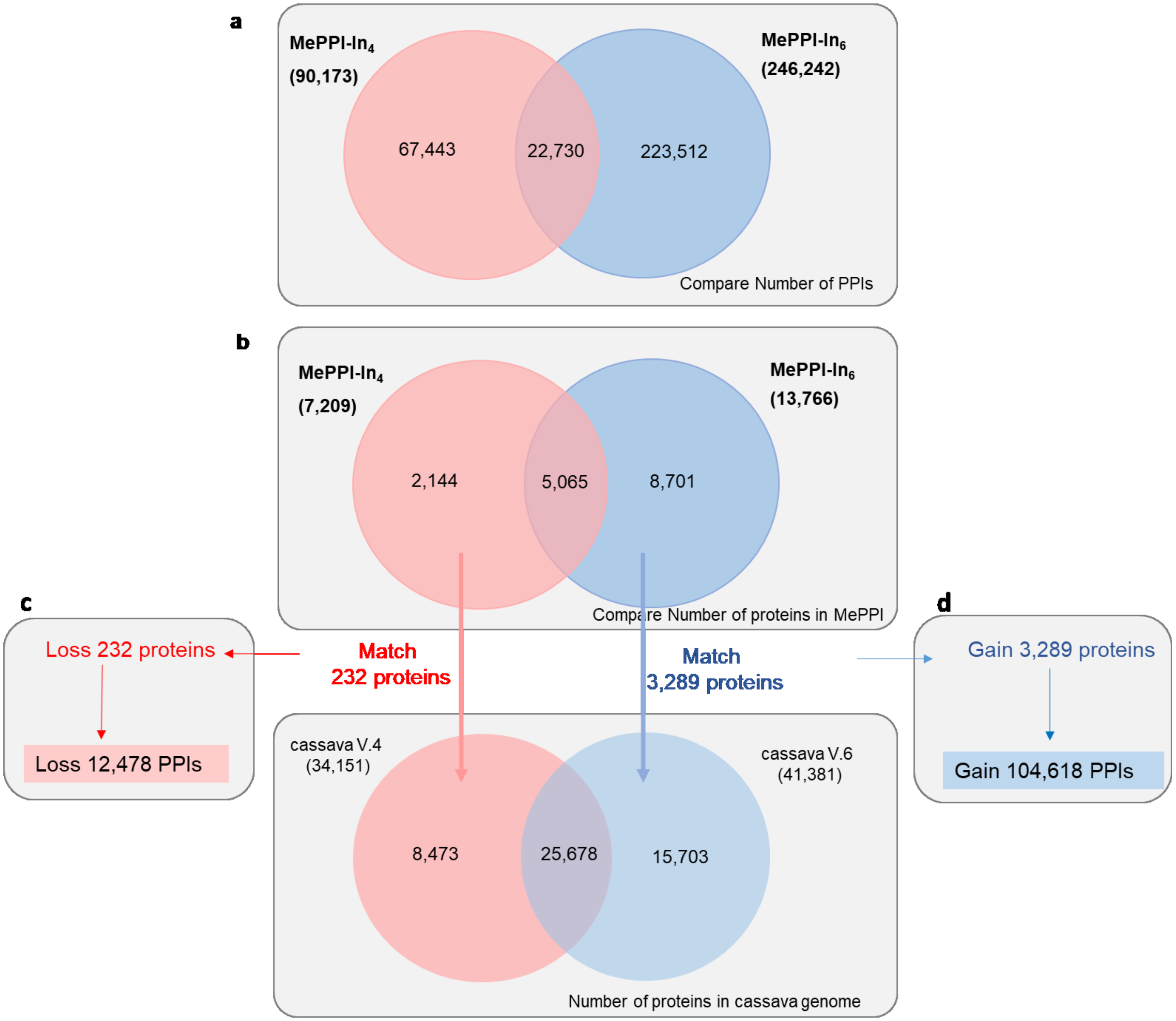

Figure S2 Comparison of MePPI-In_4[56] and MePPI-In_6. (a) represented number of PPIs from MePPI-In_4[56] and MePPI-In_6. (b) showed number of proteins in MePPI-In_4[56] and MePPI-In_6, which could match to unique proteins in cassava V.4 (232 proteins) and cassava V.6 (3,289 proteins). (c) and (d) showed number of PPIs in MePPI-In_6 which were lost and gained PPIs based on update genome version, respectively. The number of unique proteins in cassava V.4, V.6 and their interactions were presented.

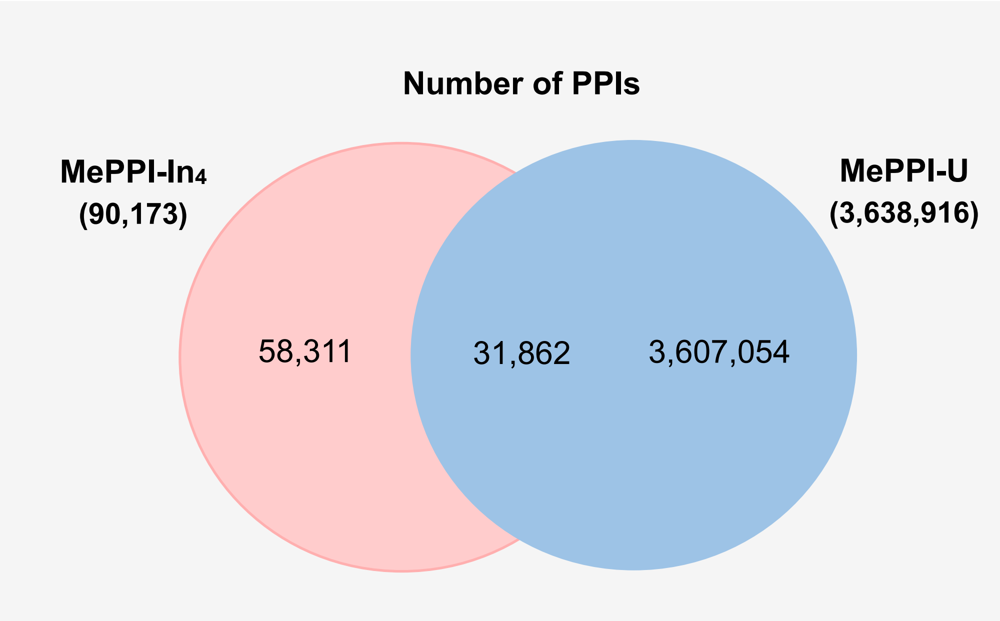

Figure S3 Comparison of cassava PPI networks from previous work ($MePPI\text{-}In_4$)[56] and current work (MePPI-U) based on PPIs comparison.

a

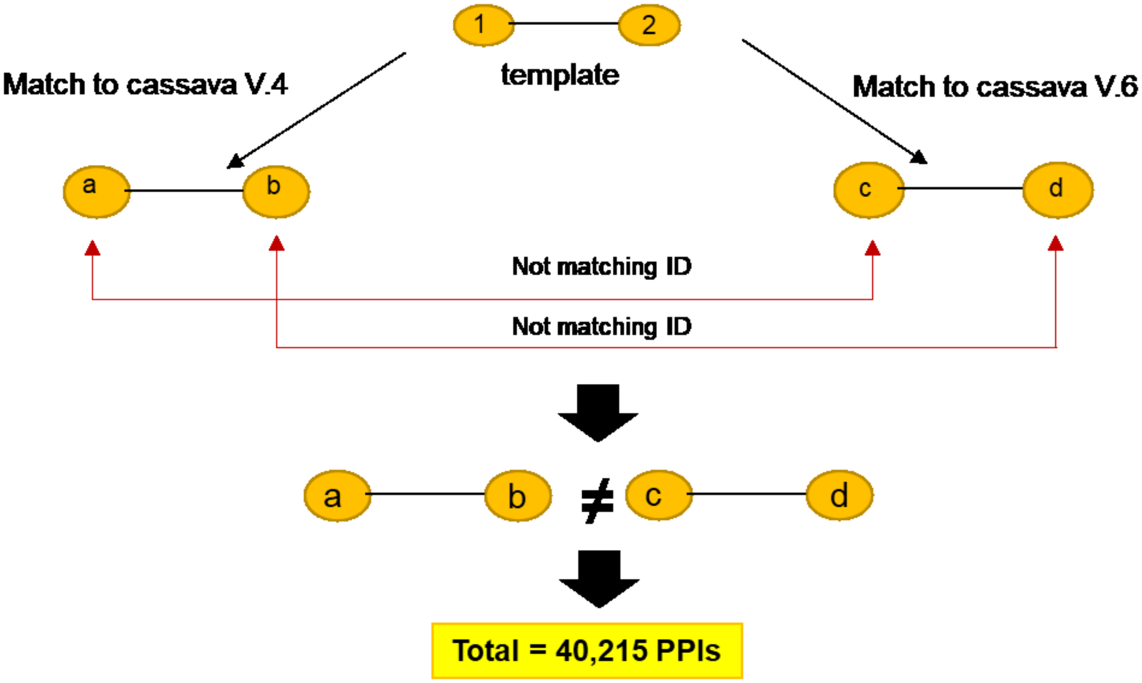

b

Template Arabidopsis	Cassava V4	Cassava V6	Cassava V4 match V6
AT4G10430.3	cassava4.1_016592m	Manes.01G000300.1.p	Manes.01G000300.2.p
AT1G33170.1	cassava4.1_003485m	Manes.01G000400.1.p	Manes.01G000400.2.p
AT5G47610.1	cassava4.1_028743m	Manes.01G000500.1.p	Manes.01G000500.1.p
AT1G10830.1	cassava4.1_009948m	Manes.01G001200.1.p	Manes.01G001200.1.p
AT2G41060.2	cassava4.1_006250m	Manes.08G067200.1.p	Manes.01G001600.1.p
AT1G33250.1	cassava4.1_004846m	Manes.01G001800.1.p	Manes.01G001800.2.p

Figure S4 Comparison of template, cassava V.4 and cassava V.6 proteins. (a) showed the schematic of matching protein from interolog-based method. Sequence re-annotation affects the homology-based analysis; updating the cassava genome from cassava V.4 to V.6 caused a mismatch of some template proteins with their counterparts in cassava, resulting in the loss of at least 40,215 putative PPIs. (b) showed the example of protein ID from template Arabidopsis, cassava V.4, cassava V.6 and cassava V.6 which were converted from cassava V.4. The color boxes represented comparison of cassava V.6 and cassava V.6 which were converted from cassava V.4; same protein ID (green) and different protein ID (red).

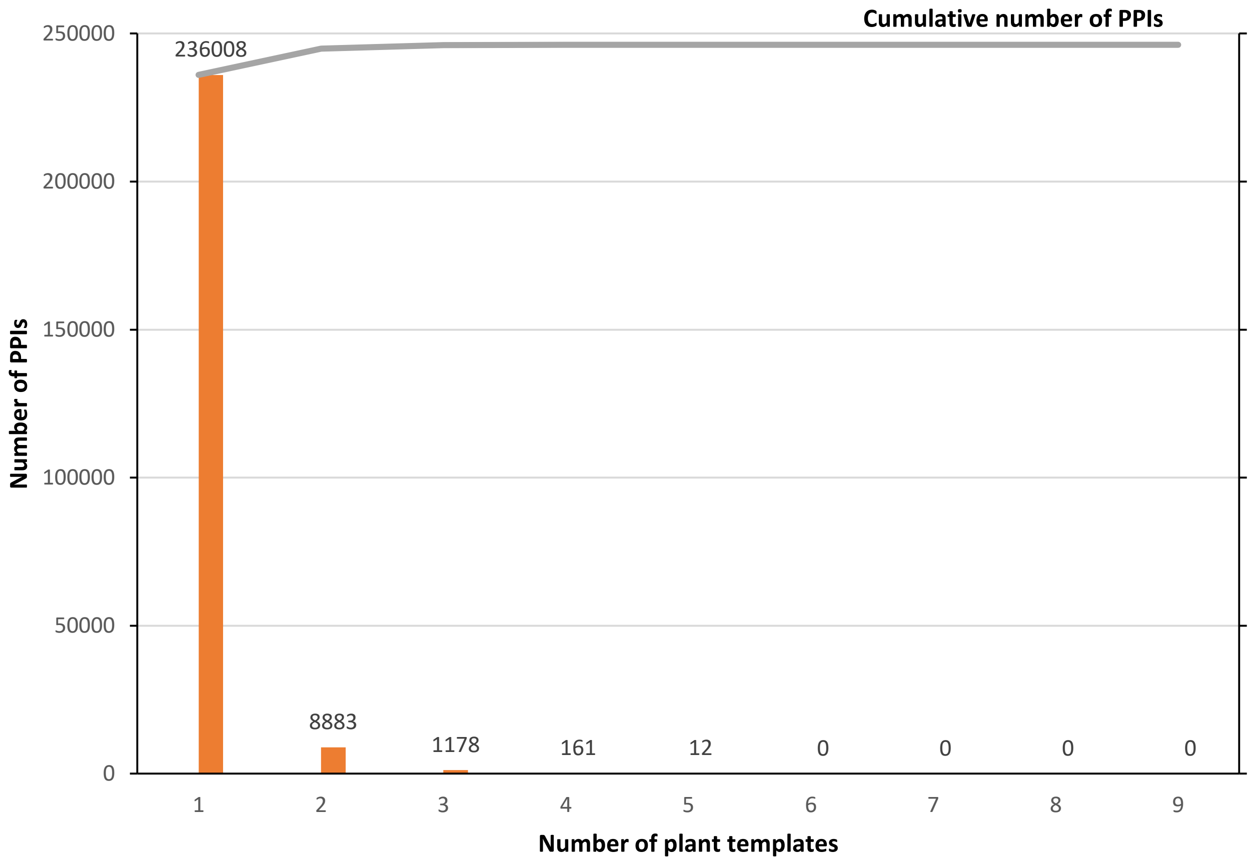

Figure S5 Number of PPIs based on number of plant templates

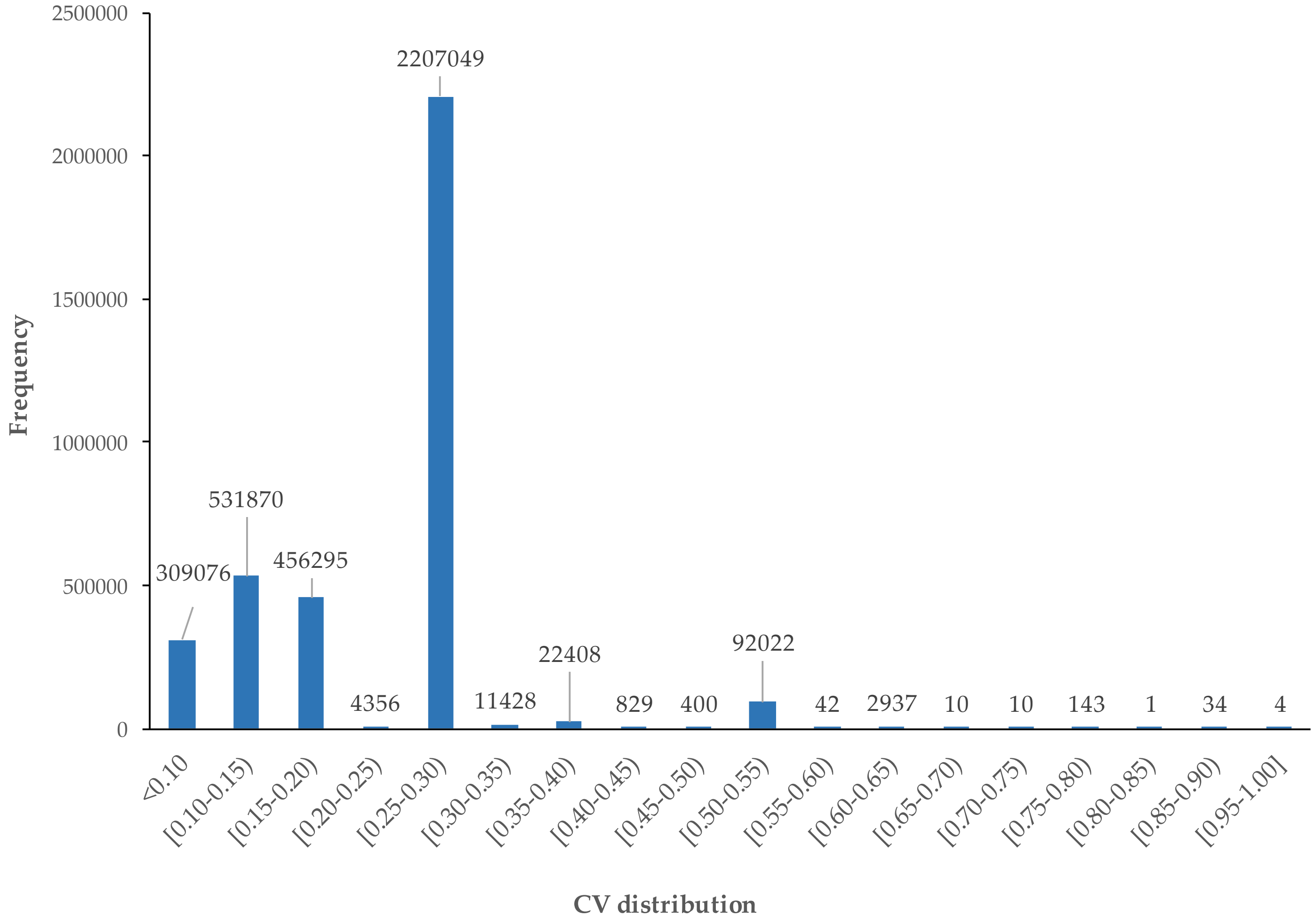

Figure S6 Distribution of the CV score of predicted PPIs in MePPI-U. X-axis represented range of CV score and Y-axis represented number of interactions.

Network properties	MePPI-U	MePPI-In$_4$[56]	Arabidopsis PPI network[22]	Rice PPI network[23]
**Dimension of network**				
- Number of nodes	24,590	7,209	11,816	5,049
- Number of edges	3,638,916	90,173	96,816	76,585
**Scale-free property**<br>power-law distribution<br>$P(k) \sim k^{-\gamma}$	$y = 1488.6x^{-0.192}$<br>$r^2 = 0.83$<br>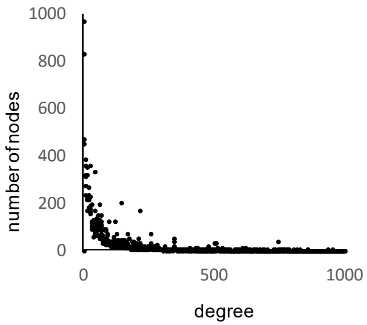 	$y = 6788.1x^{-1.534}$<br>$r^2 = 0.90$<br>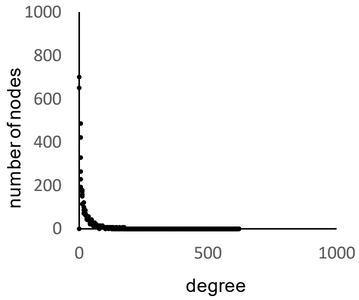 	$y = 17188x^{-1.738}$<br>$r^2 = 0.92$<br>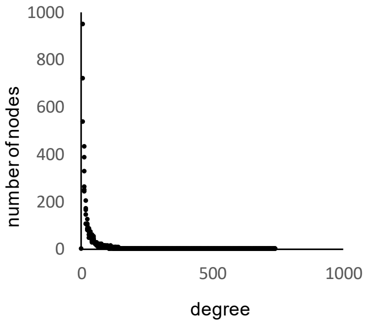 	$y = 1993.7x^{-1.277}$<br>$r^2 = 0.86$<br>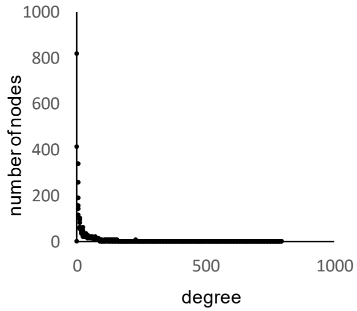

Figure S7 The global network properties of protein-protein interaction networks in cassava (MePPI-U, MePPI-In$_4$[56]), Arabidopsis[22] and rice[23].

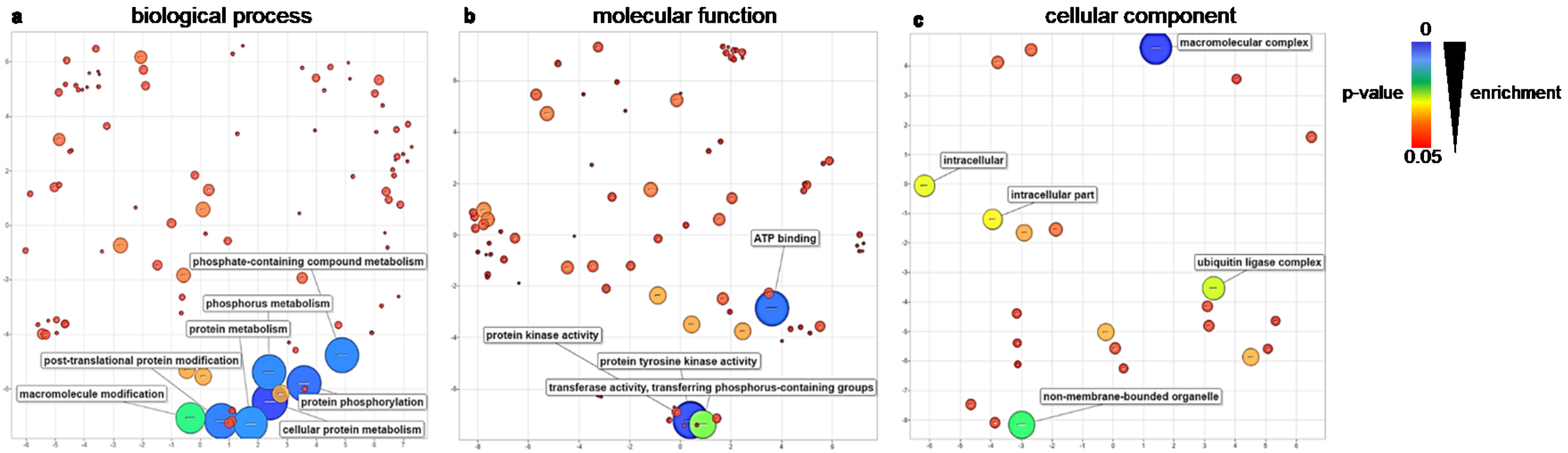

Figure S8 The functional content of protein in MePPI-U. The scatterplots were created based on GO enrichment analysis. The node color showed degree of enrichment ranging from low (red) to high (blue), while the node size represented the frequency of the proteins in each functional group
